# Multipotent Effect of Clozapine on Lipopolysaccharide-Induced Acetylcholinesterase, Cyclooxygenase-2,5-Lipoxygenase, and Caspase-3: In Vivo and Molecular Modeling Studies

**DOI:** 10.3390/molecules30020266

**Published:** 2025-01-11

**Authors:** Minhajul Arfeen, Devendra Kumar Dhaked, Vasudevan Mani

**Affiliations:** 1 Department of Medicinal Chemistry and Pharmacognosy, College of Pharmacy, Qassim University, Buraydah 51452, Saudi Arabia; 2 Department of Pharmacoinformatics, National Institute of Pharmaceutical Education and Research (NIPER)-Kolkata, Kolkata 700054, India; dkdhaked@niperkolkata.edu.in; 3 Department of Pharmacology and Toxicology, College of Pharmacy, Qassim University, Buraydah 51452, Saudi Arabia; v.samy@qu.edu.sa

**Keywords:** clozapine, molecular docking simulation, virtual screening, inflammation, neurodegenerative disorder, diabetes, Alzheimer’s diseases

## Abstract

Dual inhibition of cyclooxygenase-2 (COX-2) and lipoxygenase (LOX) is a recognized strategy for enhanced anti-inflammatory effects in small molecules, offering potential therapeutic benefits for individuals at risk of dementia, particularly those with neurodegenerative diseases, common cancers, and diabetes type. Alzheimer’s disease (AD) is the most common cause of dementia, and the inhibition of acetylcholinesterase (AChE) is a key approach in treating AD. Meanwhile, Caspase-3 catalyzes early events in apoptosis, contributing to neurodegeneration and subsequently AD. Structure-based virtual screening of US-FDA-approved molecules from the ZINC15 database identified clozapine (CLOZ) as the dual inhibitor of COX-2 and AChE, with significant binding affinity. Further molecular docking of CLOZ in the active site of LOX and Caspase-3 also showed significant binding potential. Further, the results from molecular docking were validated using molecular dynamics simulation (MDS) studies, confirming the results from molecular docking. The results from MDS showed good binding potential and interactions with key residues. The CLOZ was further assessed using lipopolysaccharide (LPS)-challenged rats treated for thirty days at doses of 5 and 10 mg/kg, p.o. The results demonstrated modulation of COX-2, 5-LOX, AChE, Caspase-3, and MDA in LPS-induced brains. Additionally, the expression level of IL-10 was also measured. Our results showed a significant decrease in the levels of COX-2, 5-LOX, AChE, Caspase-3, and MDA. Our results also showed a significant decrement in the pro-inflammatory markers NF-κB, TNF-α, and IL-6 and an improvement in the levels of anti-inflammatory markers IL-10 and TGF-β1. Overall, the findings indicate that CLOZ has potential for neuroprotective effects against LPS-treated rats and can be explored.

## 1. Introduction

Dementia is a disorder characterized by a significant decline from one’s previous level of cognition and is the seventh leading cause of death among all diseases. As per the report published by the WHO in the year 2024, over 55 million people are living with dementia across the globe, and around 10 million new cases emerge annually. This number is projected to increase up to 78 and 139 million by the years 2030 and 2050, respectively [1]. Alzheimer’s disease (AD) is the most common cause of dementia. The economic burden of managing AD in the U.S. is substantial, with care costs estimated at USD 305 billion in 2020, rising to USD 360 billion by 2024. Projections indicate these costs could surpass USD 1 trillion by 2050, reflecting the disease’s growing prevalence and healthcare demands [2]. Cholinergic transmission plays an important role in central nervous system functions, particularly learning and memory. Brains with dementia with characterized by elevated levels of inflammation, and recent findings demonstrate COX-2 in the center of the inflammatory response. Numerous studies have documented Caspases-mediated apoptosis as an early event in AD. The activation of Caspase not only contributes to neuronal cell death, but also facilitates the underlying etiology of the disease. AD is accompanied by oxidative stress, and malonaldehyde (MDA) is a very well-documented bio-marker for oxidative stress. Lipoxygenases (LOX), particularly 5-LOX, have been documented to promote neuroinflammation, oxidative stress, and amyloid-beta (Aβ) and disrupt neuronal and vascular integrity [3,4]. Inhibition of the above-mentioned targets along with AChE is a well-established and promising strategy for the treatment and prevention of AD.

**Figure 1:**
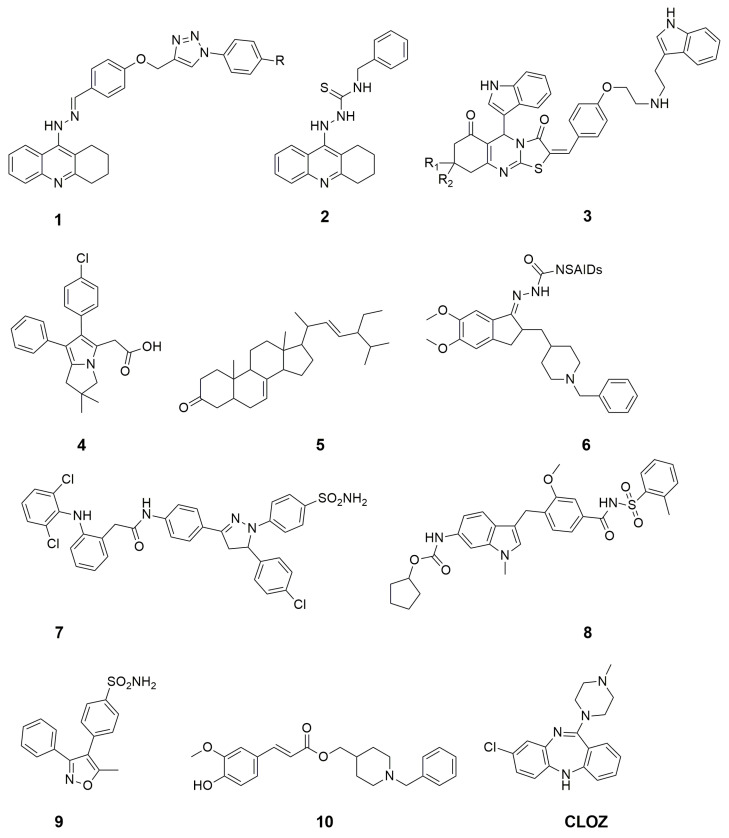
Chemical structures of the few ligands with multi-target action.

AD is a multifactorial disease, i.e., under diseased conditions, several molecular targets are either upregulated or downregulated [5]. In this context, several reports were published that displayed multi-target action for improvement of AD. For example, Al-Fadly et al. reported Tetrahydroacridine-triazole (**1**, Figure 1) and thiosemicarbazide (**2**) hybrids as COX-2, 15-Lipooxygenase (LOX) hBChE, and hAChE inhibitors to serve as multi-target directed ligands for AD treatment [6]. Javed et al. performed structural modifications on pyrimidine and pyrrolidone cores for the inhibition of COX-2, 5-LOX, BChE, and AChE [7]. Nadeem et al. reported indole derivatives carrying 2-arylidene derivatives of thiazolopyrimidine (**3**) as multitarget inhibitors of monoamine oxidase (MAO)-A, MOA-B, BChE, and AChE [8]. Martinez et al. evaluated terpenes from industrial orange juice by-products against the inhibition of LOX, BChE, and AChE for AD [9]. Razavi et al. reviewed the potential of licofelone (**4**, an NSAID) for the treatment of neurological disorders including AD. It is relevant to mention that licofelone is an established COX and LOX inhibitor [10]. Alqahtani et al. extracted stigmasta-7,22-diene-3-one (**5**) from Isodon rugosus for the inhibition of COX, LOX, BuChE, and AChE [11]. Fang et al. reported donepezil coupled with conventional NSAIDs (**6**) for the inhibition of COX-1, COX-2, BuChE, and AChE [12]. Javed et al. reported diclofenac derivatives (**7**) as concomitant inhibitors of AChE, MAO, COX-2, and 5-LOX for the potential agents against AD [13]. Kalra et al. demonstrated that modulation of COX and LOX inflammatory pathways provides mitoprotection to AD rats using Zafirlukast (**8**) and valdecoxib (**9**) [14]. Dias et al. reported a hybrid of feruloyl-donepezil (**10**) as the multi-target inhibitory ligand for COX-1, COX-2, 5-LOX, and AChE [15]. Zhang et al., while reviewing the nine important strategies for improved treatment of AD, highlighted the importance of dual inhibition of COX and LOX [16]. Similarly, Cornec et al. reviewed multitargeted imidazoles as potential therapeutic leads for AD and other neurodegenerative diseases and outlined the importance of COX and LOX pathway modulation in improved AD treatment [17].

**Figure 2:**
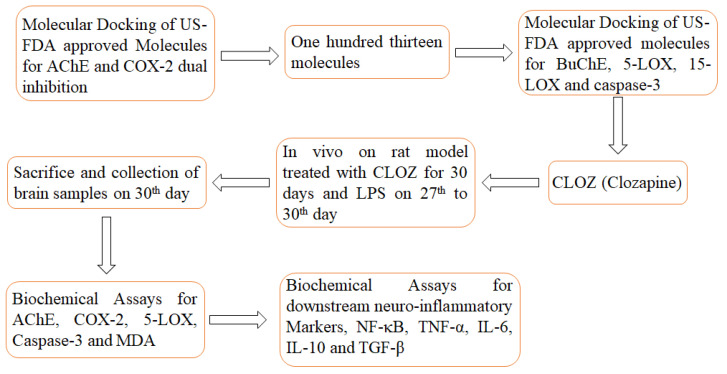
Workflow utilized in this report.

Clozapine (CLOZ) is an FDA-approved atypical tricyclic anti-psychotic molecule used for the treatment of schizophrenia. However, its use is limited due to the associated agranulocytosis and toxic hepatitis in a few cases. Because clozapine displays a promising therapeutic profile, it can be used as a template to develop new chemical entities devoid of agranulocytosis. Repurposing existing drugs, including those derived from chemically diverse natural products, presents a cost-effective and efficient strategy to address unmet medical needs. The inherent chemo-diversity of natural products, with their unique structural complexity and functional variability, serves as a valuable resource for identifying new therapeutic applications. Similarly, by leveraging the established pharmacodynamics and pharmacokinetics profile of known molecules, this approach can significantly accelerate the drug development pipeline [18,19]. In this work, structure-based virtual screening of US-FDA-approved molecules from the ZINC15 database identified CLOZ as a dual inhibitor of COX-2 and AChE. Further molecular docking was performed into the active sites of 5-LOX, 15-LOX, and Caspase-3. The binding potential of CLOZ against the above-mentioned proteins was also evaluated using molecular dynamics simulation (MDS), followed by biological evaluation using in vivo model. As AD is characterized by inflammation oxidative stress, clozapine was also evaluated for downstream inflammatory markers like NF-κB, TNF-α, IL-6, IL-10, and TGF-β1. To the best of our knowledge, the above-mentioned molecular targets are not very well documented for CLOZ. This study aims to provide additional evidence that COX-2, 5-LOX AChE, and Caspase-3 are molecular targets and can be modulated by the first atypical antipsychotic CLOZ discovered in the year 1958. Figure 2 shows the workflow taken up in this report.

## 2. Results

### 2.1. Molecular Docking

The molecular docking of US-FDA-approved molecules obtained from the ZINC15 database were docked into the active sites of AChE and COX-2. The results identified CLOZ as a promising dual inhibitor of both AChE and COX-2. The CLOZ was further docked into the active sites of BuChE, 5-LOX, 15-LOX and Caspase-3. The CLOZ demonstrated notable binding affinities with AChE and BuChE, registering binding scores around 9.1 and 9.2 kcal/mol, respectively, as shown in Table 1. These scores were comparable to the co-crystallized ligands for AChE and COX-2, which had affinities of approximately 12.1 and 10.1 kcal/mol. Besides cholinesterases AChE and BuChE, CLOZ was also docked into the active sites of COX-2, 5-LOX, 15-LOX, and Caspase-3. The predicted binding affinities of CLOZ against the above-mentioned enzymes were 7.9, 7.6, 8.8, and 7.4 kcal/mol, respectively. The predicted binding affinities of the co-crystallized ligand for the aforementioned enzymes were 9.1, 9.7, 5.8, and 8.2 kcal/mol, respectively. Analysis of CLOZ’s low-energy conformations indicated that its most stable binding poses interacted with key residues across multiple docked complexes.

### 2.2. Molecular Dynamics

MDS is crucial for exploring the dynamic interactions within protein–inhibitor complexes, revealing stability, binding interactions, and conformational flexibility. Unlike static docking, MD captures how temperature, solvent, and pressure affect molecular movements over time. This approach provides detailed insights into transient interactions such as hydrogen bonds and hydrophobic contacts, which are essential for understanding protein inhibition and enhancing inhibitor design in drug discovery. Therefore, in this work, the CLOZ complexed with AChE, BuChE, COX-2, 5-LOX, 15-LOX, and Caspase-3 was considered for MDS to evaluate their stability and binding interaction under dynamical conditions.

#### 2.2.1. Root Mean Square Deviations

**Figure 3:**
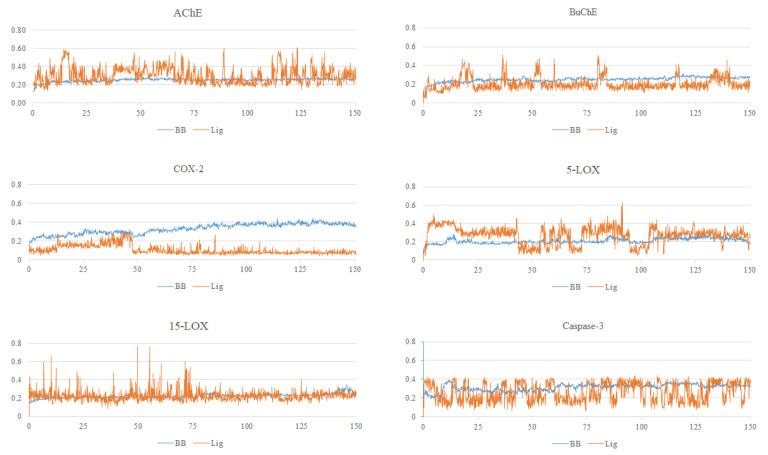
RMSD plots for CLOZ complexed with AChE, BuChE, COX-2, 5-LOX, and 15-LOX. The *x*-axis represents the simulation time in nanoseconds (ns), and the *y*-axis represents RMSD in nanometers (nm).

Figure 3 and Table 2 show RMSD values and the average RMSD values calculated over 150 ns of a simulation run for the complexes CLOZ complexed with AChE, BuChE, COX-2, 5-LOX, 15-LOX, and Caspase-3. The average RMSD value of backbone atoms across all the complexes varied in the range of 0.21 to 0.33 Å, while the standard deviation value varied in the range of 0.02 to 0.06 Å. Similarly, the average RMSD value for CLOZ varied in the range of 0.11 to 0.33 Å, and the standard deviation value for the calculated RMSD’s of ligand was in the range of 0.05 to 0.09 Å. These low values of RMSDs across all complexes indicate that the backbone structure remained close to the reference conformation across the simulation run. Similarly, the low value of the standard deviation indicates minimal fluctuations around the average RMSD values, thus further consolidating the stability of the backbone in all complexes. Similarly, the low average RMSD and standard deviation values of the ligand in all complexes indicates good stability of the CLOZ across all enzyme complexes.

#### 2.2.2. Radius of Gyration

**Figure 4:**
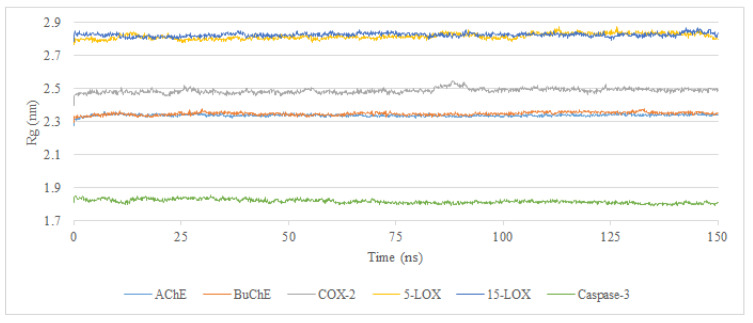
Radius of gyration (Rg, nm) for all proteins complexed with CLOZ.

To assess the stability of the CLOZ–protein complexes, the radius of gyration (Rg) was used. A significant persistent Rg value for the protein inhibitor complex over the simulation period indicates that the overall shape and size of the protein were unaffected upon ligand binding. This could further be interpreted as proving that the binding event did not cause any significant structural folding rearrangements or unfolding processes within the protein. The persistent value of Rg may further imply that the protein structure was inherently robust or that the ligand binding took place without inducing any major conformational changes. For all complexes, the values ranged from 1.8 to 2.85 nm, with standard deviations between 0.01 and 0.02 nm, suggesting minimal fluctuation around the mean. Additionally, the standard error of the mean was between 0.0002 and 0.0004 nm, further confirming the consistency of values and indicating stable protein structures in the presence of CLOZ (Table 3). Figure 4 shows the variation in the Rg for all protein complexes calculated for 150 ns of a simulation run.

#### 2.2.3. Root Mean Square Fluctuation (RMSF)

**Figure 5:**
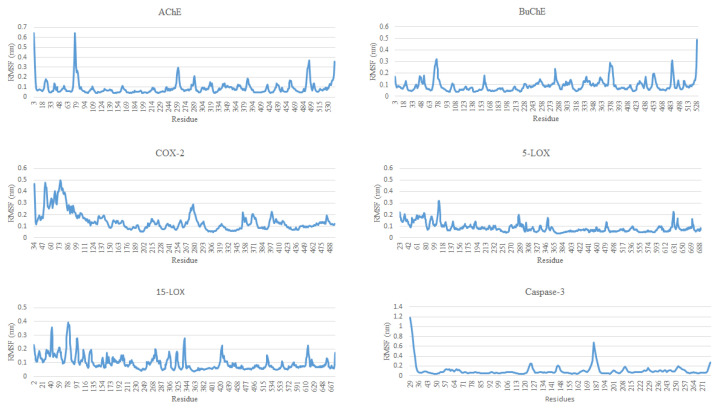
Root mean square fluctuations for residues of all the complexes.

RMSF is a commonly used metric in molecular dynamics (MD) simulations to quantify the flexibility of individual residues within a protein. RMSF represents the average deviation of each residue or atom from its mean position over the course of the simulation, indicating the extent of fluctuations in different regions of the molecule. Higher RMSF values typically correspond to flexible regions, such as loops or surface-exposed areas, while lower values suggest more rigid, structurally stable regions, often in the protein’s core. RMSF analysis helps to pinpoint flexible and stable areas, providing insights into the protein’s structural dynamics and functional sites. The RMSF values for all proteins in consideration varied in the range of 0.2 to 0.5 nm. For AChE, the RMSF values showed significant fluctuations for residues at the N-terminal region, with the peak of residue 43 reaching 3.5 nm. Beyond residue 43, the RMSF value stabilized around 1 nm with minor fluctuations at the C terminal, indicating that the N-terminal of the AChE is highly flexible, with the rest of the protein being relatively stable. For BuChE, the RMSF values were lower and remained below 0.5 nm. Periodic spikes were observed, indicating the regions of flexibility, especially towards the C-terminal end of the protein. Overall, the BuChE displayed uniform stability, with minor fluctuations throughout the protein. For the complex of COX-2, the fluctuations were observed to be marginally higher at the N-terminal regions, with residues around 72 displaying values of 0.5 nm. Beyond residue 72, the RMSF values decreased to 0.2 nm, with peaks scattered across the protein suggesting that the N-terminal region of the COX-2 is marginally flexible while the rest of the protein is stable. For 5-LOX, the RMSF fluctuations for most of the residues stayed around 0.2 nm, with residue 133 displaying highest the RMSF value of 0.35 nm, in addition to the few minor peaks that were observed throughout the protein structure. Overall, the 5-LOX displayed general stability across the protein. The residues of 15-LOX displayed moderate fluctuations, with residues 22, 292, and 372 displaying the highest RMSF values of 0.4 nm. As observed for 5-LOX, most of the fluctuations were minor, suggesting stable regions with flexible areas in between. Overall, AChE displayed the highest flexibility among the proteins, particularly at the N-terminus, while COX-2, 5-LOX, and 15-LOX showed more moderate fluctuations. BuChE appeared to be the most stable, with fewer regions of high flexibility. Figure 5 shows the RMSFs for all complexes.

#### 2.2.4. Binding Energy

**Figure 6:**
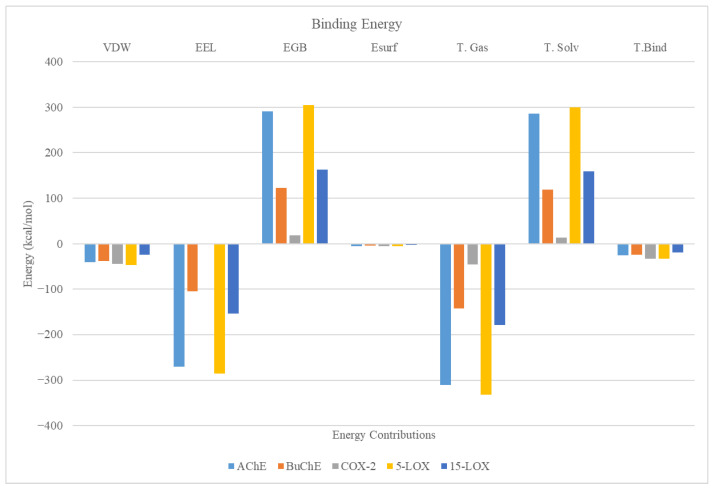
Individual contributions to the binding energy for all complexes taken up for MDS.

Binding energy is a critical parameter that quantifies the strength and stability of the complex formed between a receptor and its ligand. Figure 6 shows the binding energy calculated for the CLOZ complexed with the AChE, BuChE, COX-2, 5-LOX, and 15-LOX using the Generalized Born Surface Area (GBSA) approach, a popular method in MDS. The gas phase energy (∆E (gas)) includes contributions from Van der Waals (∆E_VDW_) and electrostatic energy (∆E_EL_) and represents energies from the direct interaction of protein and CLOZ in the absence of solvent. The solvent phase energy (∆E_solv_) is split into polar solvation energy (∆E_GB_) and non-polar solvation energy (∆E_surf_). ΔE_Bind_ represents the net binding energy, combining both gas-phase and solvent-phase energy to predict the overall favorability of the protein–CLOZ binding. It should be noted that the binding energies shown in this work do not include entropy contributions and, hence, represent the enthalpy contributions to binding free energy. The total binding energy of CLOZ for all complexes varied in the range of ~8 to ~32 kcal/mol, with COX-2 and 5-LOX displaying the highest binding energy of 32 kcal/mol. The binding energy of CLOZ for cholinesterase was found to be ~25 kcal/mol. The binding energy of CLOZ for Caspase-3 was the lowest, with a value of ~8 kcal/mol. The individual contributions to the binding energy were also calculated. The gas-phase VDW energies were negative across all the complexes, indicating favorable hydrophobic interactions. The gas-phase EEL values were strongly negative for all complexes except for COX-2 and Caspase-3, indicating significant electrostatic interactions across AChE, BuChE, 5-LOX, and 15-LOX. A high amount of polar solvation energy was noted across all the enzymes except COX-2 and Caspase-3, indicating significant resistance from the solvent molecules during the binding of CLOZ with AChE, BuChE, 5-LOX, and 15-LOX. The non-polar solvation energy was found to be marginally negative across all the complexes, indicating minor favorable contributions to the binding energy from solvation of the ligand. The gas-phase energy was found to be a highly favorable protein–ligand interaction. The solvent-phase energy was found to highly unfavorable, except for COX-2 and Caspase-3. Overall, the binding of CLOZ to AChE, BuChE, 5-LOX, and 15-LOX was driven by both by electrostatic and VDW despite a high amount of solvation penalties. The binding of CLOZ to COX-2 and Caspase-3 was mainly driven by hydrophobic interactions. Further, the CLOZ displayed the highest amount of binding energy for COX-2 and 5-LOX.

**Figure 7:**
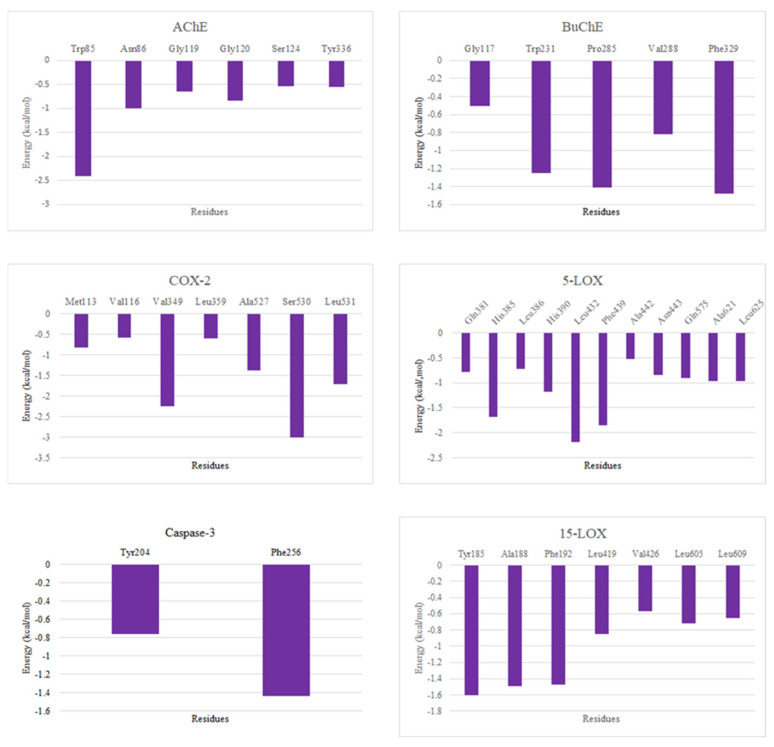
Residue contributions to the binding energy in all six complexes.

**Figure 8:**
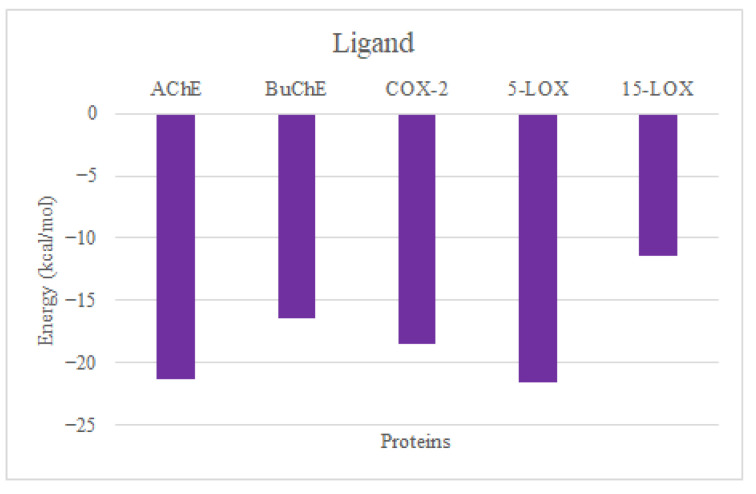
CLOZ contributions to the binding energy in all six complexes.

Figure 7 presents the per-residue energy decomposition analysis for the interactions of ligands with AChE, BuChE, COX-2, 5-LOX, and 15-LOX. The results indicate significant contributions from specific residues to the binding energy across all targets. For AChE, Trp85 exhibited the strongest interaction, contributing approximately −2.5 kcal/mol, while Asn86 and Gly120 provided moderate stabilization (~1.0 kcal/mol). In BuChE, Phe329, Pro285, and Trp231 were the major contributors, with energies around −1.6, −1.4, and −1.2 kcal/mol, respectively. For COX-2, Ser530 displayed the highest contribution at approximately −3.0 kcal/mol, followed by Val349 (−2.2 kcal/mol), Leu531 (−1.7 kcal/mol), and Ala527 (−1.4 kcal/mol). The interaction profile of 5-LOX showed notable contributions from residues His385, Leu432, and Phe439, with energy values approximately −2.0 kcal/mol. Additionally, Gln381, His390, Asn443, Gln575, Ala621, and Leu625 made notable contributions (~−1.0 kcal/mol). In 15-LOX, Tyr185, Ala188, and Phe192 were the most significant contributors, with interactions around −1.6 kcal/mol. In Caspase-3, Tyr204 and Phe256 made notable contributions. The contribution of the ligand (Figure 8) to the binding energy across all proteins reveal the strongest affinity towards AChE and 5-LOX kcal/mol, followed by COX-2 and BuChE, with values above ~−20.00 and −15 kcal/mol, respectively. The ligand contributions to the binding energy of 15-LOX and Caspase-3 were found to be ~−12 and −5 kcal/mol. These insights highlight critical residues driving ligand binding, providing a basis for rational modifications to improve specificity and binding efficacy.

### 2.3. Biochemical Analysis

**Figure 9:**
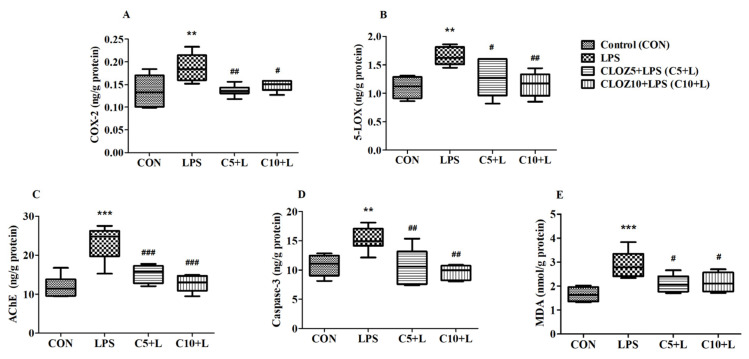
Effect of clozapine (CLOZ) on (**A**) COX-2, (**B**) 5-LOX, (**C**) AChE, (**D**) Caspase-3, and (**E**) MDA in LPS-induced rats. The results are expressed by mean ± SEM (*n* = 6). One-way ANOVA followed by Tukey–Kramer multiple comparisons test. ** *p* < 0.01 and *** *p* < 0.001 vs. control group; # *p* < 0.05, ## *p* < 0.01, and ### *p* < 0.001 vs. LPS-induced group.

Figure 9 shows the effects of CLOZ on various biochemical markers related to inflammation, oxidative stress, and cell death in LPS-induced rats. These markers include COX-2, 5-LOX, AChE, Caspase-3, and MDA. The figure displays data for four groups—Control, LPS, and CLOZ + LPS—at two different doses (C5 + L and C10 + L for 5 and 10 mg/kg, respectively). Statistical significance was assessed by one-way ANOVA followed by Tukey–Kramer multiple comparisons. The results showed that COX-2 levels were significantly increased in the LPS group compared to the control group (*p* < 0.01), indicating an inflammatory response induced by LPS. CLOZ treatment at both doses significantly reduced COX-2 levels compared to the LPS group (*p* < 0.01 for C5 + L and *p* < 0.05 for C10 + L), suggesting that CLOZ can mitigate the LPS-induced upregulation of COX-2. Similar to COX-2, 5-LOX levels were also significantly high in the LPS group (*p* < 0.01 vs. control), reflecting enhanced inflammation. CLOZ treatment significantly lowered 5-LOX levels (*p* < 0.05 for C5 + L and *p* < 0.01 for C10 + L vs. LPS), indicating that CLOZ also reduced 5-LOX levels, with the higher dose demonstrating a more substantial effect. As observed for COX-2 and 5-LOX, AChE levels were significantly increased in the LPS group compared to the control group (*p* < 0.001), suggesting potential neuroinflammatory or neurotoxic effects of LPS. Both doses of CLOZ significantly decreased AChE levels (*p* < 0.001 for C5 + L and C10 + L vs. LPS), indicating that CLOZ can counteract LPS-induced AChE upregulation, with the higher dose showing a stronger effect. Caspase-3 levels, a marker of apoptosis, were also significantly higher in the LPS group compared to the control (*p* < 0.01), suggesting that LPS induces cell death pathways. Treatment with CLOZ significantly reduced Caspase-3 levels (*p* < 0.01 for C5 + L and C10 + L), highlighting CLOZ’s potential anti-apoptotic effect, particularly at the higher dose. MDA, a marker of lipid peroxidation and oxidative stress, was significantly increased in the LPS group compared to the control (*p* < 0.001), indicating oxidative damage induced by LPS. CLOZ treatment reduced MDA levels (*p* < 0.05 for C5 + L and C10 + L), demonstrating CLOZ’s role in mitigating oxidative stress, with the higher dose again showing a greater effect.

**Figure 10:**
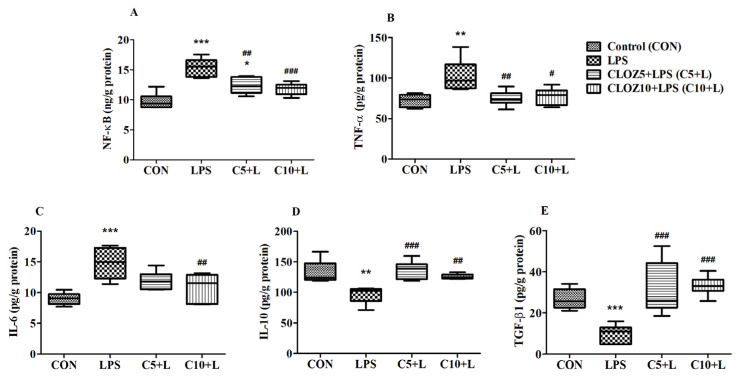
Effect of clozapine (CLOZ) on neuroinflammatory parameters: (**A**) NF-κB, (**B**) TNF-α, (**C**) IL-6, (**D**) IL-10, and (**E**) TGF-β1 in LPS-induced rats. The results are expressed by mean ± SEM (*n* = 6). One-way ANOVA followed by Tukey–Kramer multiple comparisons test. * *p* < 0.05, ** *p* < 0.01, and *** *p* < 0.001 vs. control group; # *p* < 0.05, ## *p* < 0.01, and ### *p* < 0.001 vs. LPS-induced group.

Figure 10 shows the effects of CLOZ on neuroinflammatory-related parameters in LPS-induced rats. The data are presented as mean ± SEM for various pro- and anti-inflammatory markers, including NF-κB, TNF-α, IL-6, IL-10, and TGF-β1, across four groups. NF-κB levels were significantly elevated in the LPS group compared to the control group (*p* < 0.001), indicating a marked inflammatory response due to LPS. Treatment with CLOZ at both doses (C5 + L and C10 + L) significantly reduced NF-κB levels compared to the LPS group (*p* < 0.01 and *p* < 0.001, respectively), suggesting that CLOZ effectively attenuated the LPS-induced activation of NF-κB, with the higher dose (C10 + L) showing a stronger reduction. LPS treatment also resulted in a significant increase in TNF-α levels (*p* < 0.01 vs. CON), another pro-inflammatory marker. CLOZ treatment at both doses significantly decreased TNF-α levels compared to the LPS group (*p* < 0.01 for C5 + L and *p* < 0.05 for C10 + L). This decrease indicated the anti-inflammatory action of CLOZ on TNF-α expression in LPS-induced neuroinflammation. Similarly to NF-κB and TNF-α, the levels of another pro-inflammatory marker, IL-6, were significantly higher in the LPS group compared to the control (*p* < 0.001), consistent with an inflammatory response. Only the higher dose of CLOZ effectively reduced IL-6 levels (*p* < 0.01 for C10 + L vs. LPS), confirming its role in mitigating LPS-induced IL-6 upregulation, with the higher CLOZ dose again providing a greater reduction.

## 3. Discussion

The “one drug, one target” paradigm has long been the cornerstone of drug development, emphasizing the design of highly selective molecules to modulate a single biological target with precision. While this approach has led to significant breakthroughs in treating diseases with well-defined molecular mechanisms, it presents notable limitations when addressing complex, multifactorial conditions such as AD [20]. A network of interrelated pathological processes characterizes AD, including Aβ accumulation, tau hyperphosphorylation, oxidative stress, neuroinflammation, and synaptic dysfunction. Targeting a single molecule or pathway often fails to address the interconnected nature of these pathological drivers, leading to suboptimal therapeutic outcomes. Moreover, compensatory mechanisms within the disease network can undermine the efficacy of single-target therapies [5,16,21]. Recent clinical failures of drugs focused solely on Aβ clearance underscore these challenges and highlight the need for more holistic strategies. The intricate and progressive nature of AD necessitates approaches that account for a multifaceted disease pathology, favoring multi-target or pathway-modulating drugs over highly selective agents. Recognizing these limitations has spurred interest in the identification of multi-targeted ligands for the treatment of complex disorders like AD. Therefore, this work employed structure-based virtual screening of US-FDA-approved drugs to identify the dual inhibitor of COX-2 and AChE. The identified molecules were further docked into the active sites of BuChE, 5-LOX, 15-LOX, and Caspase-3. The molecular docking results identified CLOZ as one of the potential molecules to modulate multiple targets. Further, the docked complexes of CLOZ were subjected to MDS for stability assessment. The efficacy of CLOZ for the targeted enzymes was further evaluated using an in vivo rat model treated with CLOZ for thirty days and with LPS for four days, i.e., from the 27th to 30th days.

Molecular docking is a vital tool in early drug development, offering a cost-effective alternative to traditional high-throughput screening [22]. Advances in algorithms and scoring functions have enhanced its accuracy in predicting binding poses and affinities, making it invaluable for hit identification, lead optimization, and assessing off-target interactions [23]. Its integration with computational and experimental methods has revolutionized drug discovery, enabling the exploration of mechanisms of action and addressing complex therapeutic challenges. Our initial molecular docking results identified CLOZ as the potential dual inhibitor of COX-2 and AChE. In addition to AChE and COX-2, the CLOZ was docked into the active sites of BuChE, 5-LOX, 15-LOX, and Caspase-3. The molecular docking results against molecular targets in consideration showed binding affinities comparable to the co-crystallized ligands. The binding mode analysis revealed that lowest energy docked conformation of CLOZ interacted with key residues and, hence, were taken up for MDS for 150 ns. Parameters like RMSD, radius of gyration, RMSF, binding energy, energy contributions, and residue-wise energy contributions to the binding energy were calculated. The low average RMSD values and minimal standard deviations for both the backbone atoms and the ligand across all enzyme complexes highlight the structural stability of the CLOZ complexes. These results further emphasize that CLOZ maintains a consistent binding pose, reinforcing its potential as a stable modulator of the studied enzymes. The consistent Rg values across all CLOZ–protein complexes, with minimal fluctuations and low standard errors, underscore the structural stability of the proteins over the 150 ns simulation. These results indicate that ligand binding does not induce significant conformational changes, further supporting the inherent robustness of the protein–CLOZ complexes. The RMSF analysis highlights distinct flexibility patterns among the CLOZ–protein complexes, with AChE exhibiting the highest fluctuations, particularly at the N-terminus, while BuChE demonstrated the greatest stability across its residues. The marginally flexible N-terminal regions in COX-2, along with the generally stable profiles observed for 5-LOX and 15-LOX, suggest that these proteins maintain their structural integrity with localized flexibility that may contribute to functional adaptability. The binding energy analysis highlights that CLOZ exhibits strong and favorable binding with COX-2 and 5-LOX, driven predominantly by hydrophobic interactions and supported by significant van der Waals contributions. Despite the solvation penalties observed for AChE, BuChE, 5-LOX, and 15-LOX, the favorable gas-phase interactions, including electrostatics and van der Waals forces, ensured stable binding, underscoring the importance of these interactions in the overall binding mechanism. Further, per-residue energy decomposition analysis revealed key amino acids contributing significantly to CLOZ binding across all protein targets, highlighting their roles in stabilizing the complexes. For AChE, Trp86, Gly120, and Gly121 emerged as significant contributors to the binding energy. Notably, Trp86 is a key residue in the peripheral aromatic binding site which plays a crucial role in the interaction with small molecules and substrates. Meanwhile, Gly120 and Gly121, along with Ala204, form part of the oxyanion binding site, a critical component of the catalytic machinery involved in the hydrolysis of ACh. For BuChE, the residues Trp231, Pro285, Val288, Phe329, and Gly117 play critical roles in ligand binding. Notably, Trp231, Pro285, and Phe329 have been documented for collectively creating a complementary binding environment within the active site. This synergy is vital for effective substrate recognition, stabilization, and catalysis [24,25]. In the case of COX-2, Val349, Ala527, Ser530, and Leu531 are key contributors in the binding of CLOZ, each contributing uniquely to the function and interaction of COX-2 with substrates and inhibitors. Val349 and Leu531 shape the hydrophobic environment and define the side pocket unique to COX-2, enabling selective inhibitor binding. Ala527, with its smaller side chain compared to the corresponding residue in COX-1, expands the active site cavity, further facilitating COX-2 specificity. Ser530 plays a catalytic role and is the site of irreversible acetylation by aspirin, blocking substrate access and prostaglandin synthesis. Collectively, these residues are critical for COX-2’s enzymatic activity, substrate specificity, and inhibitor binding, making them focal points in anti-inflammatory action [24,25,26]. For the case of 5-LOX, notable contributors are His367, Leu414, Phe421, Gln363, His372, Asn425 Gln557, Ala603, and Leu607, which play crucial roles in ligand binding by forming a network of interactions that stabilize ligands and facilitate enzymatic activity. His367 and His372 are central to the catalytic mechanism, as they coordinate the non-heme iron essential for oxygenation reactions, ensuring the proper alignment of ligands with the catalytic core [27]. Leu414, Phe421, Ala603, and Leu607 contribute to the hydrophobic environment of the active site, stabilizing hydrophobic ligands such as CLOZ. Gln363, Asn425, and Gln557 are involved in forming hydrogen bonds and polar interactions, enhancing ligand specificity and stabilization. These residues play a dynamic role in orienting ligands within the active site, ensuring proper positioning for catalytic efficiency [28]. In 15-LOX, Tyr185, Ala188, and Phe192 contributed significantly to CLOZ binding and were similar to the interactions reported in previous studies [29,30]. For Caspase-3, Tyr204 and Phe256 played notable roles in the binding of CLOZ and have been reported as unique residues present in the S2 subsite of Caspase-3 [31]. The contribution of a ligand to the binding energy is crucial in determining the strength, stability, and specificity of its interaction with a target protein. This aspect is particularly important as it reflects the ligand’s ability to form strong and favorable interactions with the active-site residues. A ligand with a high contribution to binding energy enhances the overall binding affinity, which is key for achieving potent inhibition of the target. In this study, CLOZ demonstrated a significant contribution to the binding energy of AChE, BuChE, COX-2, and 5-LOX, highlighting its potential as a multitarget inhibitor. This underscores the promising role of CLOZ in modulating key enzymes associated with AD and neuroinflammation.

The dual inhibition of COX-2 and 5-LOX represents a promising therapeutic strategy for managing inflammation-driven diseases, including neurodegenerative conditions such as AD [10,17]. COX-2 and 5-LOX are critical enzymes in the arachidonic acid metabolic pathway, responsible for producing pro-inflammatory mediators like prostaglandins and leukotrienes, respectively. Chronic neuroinflammation, driven in part by these mediators, is increasingly recognized as a central factor in the pathogenesis and progression of AD. While selective COX-2 inhibitors have shown potential in alleviating neuroinflammation [32,33], their long-term use is associated with cardiovascular risks, necessitating safer alternatives. Similarly, 5-LOX inhibitors have demonstrated benefits in reducing leukotriene-mediated oxidative stress and Aβ accumulation in preclinical models of AD [7,34,35]. Dual inhibition of COX-2 and 5-LOX offers a synergistic approach by targeting both arms of the inflammatory cascade, potentially providing enhanced neuroprotection and mitigating adverse effects linked to single-target therapies [36]. Therefore, taking into consideration the results from molecular docking and dynamics, the CLOZ was evaluated for its efficacy using an in vivo rat model treated with two doses of 5 mg/kg and 10 mg/kg for thirty days and with LPS on the 27th to 30th days. Our results showed significant decreases in the levels of both the inflammatory enzymes COX-2 and 5-LOX. CLOZ’s capacity to markedly reduce COX-2 and 5-LOX expression highlights its strong anti-inflammatory potential. This property could be instrumental in suppressing the production of prostaglandins and leukotrienes, as they are major contributors to driving the inflammatory cascade.

The simultaneous modulation of COX-2 and 5-LOX by CLOZ exhibited a significant impact on key inflammatory mediators, including NF-κB, TNF-α, and IL-6, as well as anti-inflammatory mediators such as IL-10 and TGF-β1 (Figure 9 and Figure 10, Section 2.3), thereby demonstrating a broad-spectrum anti-inflammatory effect. COX-2 and 5-LOX products, such as prostaglandins (e.g., PGE2) and leukotrienes (e.g., LTB4), play essential roles in activating NF-κB [37,38], a transcription factor that regulates the expression of various pro-inflammatory genes. NF-κB activation occurs in response to inflammatory stimuli and is perpetuated by positive feedback mechanisms driven by prostaglandins and leukotrienes. The reduction in the levels of COX-2 and 5-LOX by CLOZ reduces the production of these mediators, thereby attenuating NF-κB activation. This suppression limits the transcription of downstream pro-inflammatory cytokines and enzymes, such as TNF-α, IL-6, and additional COX-2 expression, creating a cascade of reduced inflammation. The modulation of NF-κB levels also prevents chronic inflammation, a hallmark of neurodegenerative disorders like AD [39]. TNF-α is a central pro-inflammatory cytokine involved in amplifying the inflammatory response [40]. Its production is regulated by NF-κB activation and is further enhanced by the presence of leukotrienes and prostaglandins [41,42,43] Elevated TNF-α levels are implicated in neuroinflammation, contributing to neuronal damage and progression of AD [44]. By suppressing both NF-κB activation and the direct inflammatory effects of leukotrienes and prostaglandins, dual modulation of COX-2 and 5-LOX can significantly lower TNF-α levels. This reduction in TNF-α not only decreases the inflammatory burden but may also protect neurons from cytokine-induced apoptosis, offering neuroprotective benefits in AD. IL-6 is another pro-inflammatory cytokine highly regulated by NF-κB [45]. It is a key player in chronic inflammation and is often elevated in AD and other neurodegenerative diseases [46]. Prostaglandins and leukotrienes can further stimulate IL-6 production, perpetuating the inflammatory loop [43,47]. The inhibition of COX-2 and 5-LOX interrupts this loop by reducing the upstream mediators driving IL-6 expression. Lower levels of IL-6 can mitigate systemic and neuroinflammation, reducing the neuronal damage and glial activation commonly observed in AD. IL-10 is an anti-inflammatory cytokine that acts as a counter-regulator of pro-inflammatory responses [48]. While COX-2 and 5-LOX inhibition primarily targets pro-inflammatory pathways, their suppression indirectly promotes an anti-inflammatory environment that can enhance IL-10 production. This shift toward an anti-inflammatory milieu can help restore immune homeostasis and reduce chronic inflammation. The upregulation of IL-10, facilitated by the reduced inflammatory pressure, may also limit the activation of microglia and astrocytes in the brain, further contributing to neuroprotection and resolution of inflammation in AD. TGF-β1 is a multifunctional cytokine with both pro- and anti-inflammatory roles, depending on the context. In neuroinflammatory conditions, TGF-β1 often plays a protective role by promoting tissue repair, dampening excessive immune responses, and supporting neuronal survival [49]. Dual inhibition of COX-2 and 5-LOX reduces the production of inflammatory prostaglandins and leukotrienes, which can help maintain TGF-β1’s anti-inflammatory and reparative functions. This modulation supports a balanced immune response, allowing TGF-β1 to contribute to neuroprotection and recovery without exacerbating inflammation.

Caspase-3 is a critical executioner enzyme in the apoptotic pathway, playing a central role in programmed cell death [50]. Elevated expression of Caspase-3 is often linked with neuroinflammatory and oxidative stress conditions, making it a key marker of neurodegeneration in conditions like AD [51,52,53]. Chronic inflammation, driven by mediators like NF-κB, TNF-α, and IL-6, creates a pro-apoptotic environment that sustains caspase-3 activation, often leading to excessive apoptosis, TNF-α, in particular, directly activates caspase-3 through the extrinsic apoptotic pathway. The modulation of COX-2 and 5-LOX by CLOZ, leading to the reduction in inflammatory mediators such as NF-κB, TNF-α, and IL-6, can influence Caspase-3 activity by mitigating the pro-apoptotic environment typically driven by chronic inflammation. Furthermore, the upregulation of anti-inflammatory mediators like IL-10 and TGF-β1 can promote cellular survival pathways, reducing the likelihood of Caspase-3 activation. This dual regulation of inflammatory and anti-inflammatory mediators may contribute to the preservation of neuronal integrity and the attenuation of apoptosis, highlighting the therapeutic potential of CLOZ targeting COX-2 and 5-LOX in reducing Caspase-3 mediated neuronal damage in neurodegenerative diseases.

AChE plays a critical role in regulating the cholinergic system by hydrolyzing acetylcholine (ACh), a neurotransmitter essential for learning, memory, and other cognitive functions. AChE regulates the cholinergic system by hydrolyzing ACh in the synaptic cleft, terminating neurotransmission and preventing overstimulation of postsynaptic receptors. This process ensures efficient signal transmission and maintains neural homeostasis. Dysregulation of AChE activity is linked to neurodegenerative diseases like Alzheimer’s and is targeted therapeutically with AChE inhibitors to enhance cholinergic function. In AD, increased AChE activity exacerbates the cholinergic deficit, contributing to cognitive decline. Inflammation, a hallmark of AD, is intricately linked to the cholinergic system [54]. Pro-inflammatory mediators such as TNF-α, IL-6, and NF-κB activation disrupt ACh signaling by promoting neuronal damage and reducing ACh synthesis. Furthermore, inflammation upregulates AChE expression, compounding the cholinergic deficit and worsening cognitive dysfunction. The modulation of COX-2 and 5-LOX pathways by CLOZ can mitigate this inflammatory burden by suppressing key mediators while enhancing anti-inflammatory cytokines like IL-10 and TGF-β1. This reduction in inflammation not only protects cholinergic neurons, but may also help restore ACh levels by limiting the overactivity of AChE. The dual inhibition of COX-2 and 5-LOX thus represents a promising strategy to address the interplay between inflammation and the cholinergic system, potentially alleviating cognitive symptoms and slowing the progression of neurodegeneration in AD.

MDA is a key biomarker of lipid peroxidation, reflecting oxidative damage to cell membranes caused by reactive oxygen species (ROS), often elevated in neurodegenerative diseases such as AD [55]. It influences cellular integrity, mitochondrial function, and systemic inflammatory responses. MDA is a by-product of ROS attacking lipids, indicating the severity of oxidative stress. It contributes to cellular damage by forming adducts with DNA and proteins, impairing their functions and perpetuating mitochondrial dysfunction. MDA activates pro-inflammatory pathways by interacting with Toll-like receptors (TLRs) and promoting cytokine release (e.g., TNF-α, IL-6). This amplifies the oxidative–inflammatory cycle, contributing to chronic inflammation in conditions like AD [56]. Elevated MDA not only signifies oxidative damage but also contributes to neuronal dysfunction by disrupting membrane integrity and promoting apoptosis [57]. The dual inhibition of COX-2 and 5-LOX by CLOZ can mitigate oxidative stress by reducing inflammatory pathways that amplify ROS production. Additionally, anti-inflammatory mediators such as IL-10 and TGF-β1, promoted through this inhibition, may counteract oxidative damage and protect neuronal integrity. By targeting both inflammation and oxidative stress, CLOZ has the potential to lower MDA levels, providing neuroprotective effects and alleviating oxidative damage associated with AD progression.

## 4. Materials and Methods

### 4.1. Molecular Docking

Molecular docking studies were performed using previous studies. The crystal structures of target proteins, COX-2 (4EY7), AChE (5-IKR), BuChE (4TPK), 5-LOX (6N2W), 15-LOX (4NRE), and Caspase-3 (1GFW), were acquired from the Protein Data Bank (PDB), while the chemical structure of CLOZ was obtained from PubChem in SDF format and converted to PDB format using Open Babel. Molecular docking studies were conducted with AutoDock Vina (version 1.1.2, La Jolla, CA, USA) [58]. Protein and ligand preparations were carried out using AutoDock tools (ADT) from MGLTools (version 1.5.6) [59]. The protein preparation involved removing heteroatoms, water molecules, and nonpolar hydrogens, then adding polar hydrogens and Kollman charges and finally converting them to PDBQT format. Similarly, the ligand preparation included minimization using a universal force field, defining torsions, adding Gasteiger charges, and converting them to PDBQT format. The grid boxes for docking were defined in AutoGrid4 (version 4.2.6), with centers aligned to the co-crystallized ligands, and grid dimensions were set to encompass all atoms of each co-crystallized ligand. Docking simulations generated ten binding modes for each target, and the top three scoring modes, based on binding affinity in kcal/mol, were selected for detailed analysis. From these, the binding mode chosen for further discussion and MDS was based on key residues cited in previous studies. To visualize hydrogen bonds and hydrophobic interactions in the docked complexes, a free version of Discovery Studio was employed.

### 4.2. Molecular Dynamics

MDS were performed using GROMACS 2023 version 5 (KTH Royal Institute of Technology, Stockholm, Sweden). CHARMM36 (version 4.6) and generalized AMBER force field (GAFF, version 2.11) were used to prepare the topology file protein and ligand, respectively. The simulation system was prepared in a cubic water box with dimensions of 11.92885 × 11.92885 × 11.92885 nm, ensuring a minimum solute–box distance of 1.0 nm, and solvated with 161,646 TIP3P water molecules to mimic an aqueous environment. The molecular dynamics simulation was initiated with energy minimization (EM) to resolve steric clashes and optimize the system geometry. The steepest descent algorithm was employed, with the minimization halting when the maximum force fell below 1000 kJ/mol. A step size of 0.01 was used, and the process was limited to a maximum of 50,000 steps. Electrostatic interactions were modeled using the Particle Mesh Ewald (PME) method with a cutoff distance of 1.2 nm, while van der Waals interactions employed a cutoff distance of 1.2 nm as well. Periodic boundary conditions were applied in all three dimensions to mimic infinite system behavior. After energy minimization, counterions were added to neutralize the system, and an additional minimization was performed using the same steepest descent algorithm, with a cutoff of 1.0 nm for both electrostatics and van der Waals interactions. Next, the system underwent equilibration in two phases, beginning with constant volume and temperature (NVT) equilibration. The leap-frog integrator was used with a 2 fs time step over 50,000 steps, corresponding to 100 ps of simulation time. Temperature coupling was achieved using a modified Berendsen thermostat, maintaining the protein–ligand and solvent groups at 300 K. Long-range electrostatic interactions were treated using PME with a cutoff of 1.2 nm, and van der Waals interactions were modeled with the same cutoff distance. Following NVT, constant pressure and temperature (NPT) equilibration was conducted using the same integrator and time step over 100,000 steps (200 ps). The pressure was regulated using the Berendsen barostat at 1.0 bar, with an isothermal compressibility of 4.5 × 10^−5^ bar^−1^. Temperature coupling was maintained as in the NVT phase, and position restraints were applied to the protein and ligand to stabilize the structure during equilibration. Finally, the production molecular dynamics (MD) simulation was performed to collect data over 150 ns. The leap-frog integrator was used with a 2 fs time step across 75,000,000 steps. Temperature coupling was maintained at 300 K for both the protein–ligand and solvent groups, while pressure was controlled at 1.0 bar using the Parrinello–Rahman barostat. Electrostatics were modeled with PME using a 1.2 nm cutoff, and van der Waals interactions employed the same cutoff distance. Bond constraints, including those involving hydrogen atoms, were applied using the LINCS (Linear Constraint Solver) algorithm. Energies and coordinates were saved every 10 ps, ensuring detailed output for subsequent analyses. This multi-step workflow ensured a robust and reliable molecular dynamics simulation. The root mean square deviation (RMSD), radius of gyration (Rg), and root mean square fluctuations of trajectory files in the analysis were performed using in-built tools. Molecular Mechanics Generalized Born Surface Area (MMGBSA) was used to calculate the binding energy and residue-wise interaction analysis.

### 4.3. Animal Study

#### 4.3.1. Animals

Thirty adult male albino rats, each weighing between 150 and 170 g and aged 11–12 weeks, were obtained from the animal facility at the College of Pharmacy, Qassim University, KSA. The rats were divided into four groups of six and housed in cages with three rats per cage. They were kept on a 12 h light/dark cycle and had unrestricted access to food and water. A one-week acclimatization period was provided prior to the initiation of drug treatments to ensure adjustment to the laboratory setting. Ethical approval for animal use in this research was secured from the Committee of Health Research Ethics, Deanship of Scientific Research, Qassim University, under research ID 23-24-18 and grant number 2023-SDG-1-HMSRC-35893.

#### 4.3.2. Treatment and Experimental Schedule

CLOZ and lipopolysaccharide (LPS) from *Escherichia coli* (O111:B4) were sourced from Sigma-Aldrich Co. (St. Louis, MO, USA) and dissolved in a 0.09% *w*/*v* normal saline (NS) solution. To investigate CLOZ’s protective effects against LPS-induced neurotoxicity, the rats were divided into four experimental groups. In the control group (CON; Group 1), animals received daily oral NS (0.1 mL/100 mg) from days 1 to 30 and were administered four intraperitoneal (i.p.) doses of NS (1 mg/kg) from days 27 to 30 to ensure standardized treatments. Group 2 (LPS) served as the LPS-only group, receiving NS orally each day and i.p. injections of LPS (1 mg/kg) on days 27 to 30 to induce neurotoxic effects. Groups 3 (CLOZ5 + LPS) and 4 (CLOZ10 + LPS) received daily oral doses of CLOZ at 5 mg/kg and 10 mg/kg, respectively, from day 1 to day 30, paired with LPS injections on the same days as Group 2. The dosing regimens for CLOZ and LPS were taken up from the previous studies [24,60,61]. The brain tissue samples were collected for ELISA analyses on the 30th day.

#### 4.3.3. Preparation of Brain Homogenate

On day 30 of the treatment, all rats were euthanized with a combination of ketamine (100 mg/kg) and xylazine (10 mg/kg) and administered i.p. for anesthesia, followed by cervical decapitation. Brain tissues were immediately removed, cut into small sections, and rinsed thoroughly with ice-cold phosphate-buffered saline (PBS, pH 7.4) to clear any blood residues. The tissues were then weighed and homogenized in PBS on ice, maintaining a 1:9 tissue-to-PBS ratio (1 g of tissue in 9 mL PBS), using a glass homogenizer. The resulting homogenates were collected for subsequent ELISA analysis to assess specific biomarkers. Total protein concentrations in each brain homogenate were measured by the biuret colorimetric assay, following the protocol provided by Crescent Diagnostics (Jeddah, Saudi Arabia).

#### 4.3.4. Biochemical Assays

The biochemical assays in this study were conducted using specific rat enzyme-linked immunosorbent assay (ELISA) kits obtained from MyBioSource Inc. (San Diego, CA, USA). The analyzed parameters included AChE (MBS2709297); neuroinflammatory markers such as COX-2 (MBS266603), 5-LOX (MBS2602074), NF-κB (MBS453975), TNF-α (MBS162068), IL-6 (MBS2701082), IL-10 (MBS702776), and TGF-β1 (MBS824788); apoptotic protein like Caspase-3 (MBS763727); and oxidative stress marker MDA (MBS268427). The ELISA protocols employed were based on either competitive or sandwich immunoassay principles. For each assay, specific antibodies, conjugates (such as horseradish peroxidase [HRP]), and detection systems were used to quantify the respective biomolecules. Rat brain homogenates or other relevant biological samples were prepared according to the manufacturer’s instructions and stored appropriately until analysis. Standards provided with the kits were used to construct calibration curves. All reagents, including biotinylated detection antibodies and streptavidin-HRP (SABC), were prepared as per the kit instructions. Wells of the ELISA plates were allocated for samples, standards, and blanks. A fixed volume (typically 100 µL) of homogenate or standard was added to the respective wells. The plates were incubated at room temperature for a specific period, allowing for antigen–antibody binding. Post-incubation, wells were washed thoroughly with the provided washing buffer to remove unbound components. A substrate solution was added to initiate a colorimetric reaction. The reaction was stopped after the recommended time, and the developed color intensity was measured at 450 nm using a BioTek microplate reader (BioTek Instruments, Santa Clara, CA, USA). Concentrations of the analytes were determined by comparing the absorbance values to standard curves generated from known concentrations of the respective biomolecules.

#### 4.3.5. Statistical Analysis

Results of the ELISA analysis are reported as mean values with standard error mean (SEM). Statistical evaluations were carried out using one-way ANOVA, followed by the Tukey–Kramer post hoc test to detect significant differences among the groups. All statistical analyses were performed using GraphPad version 9.0 (GraphPad Software Inc., San Diego, CA, USA), with significance defined at *p* < 0.05.

## 5. Conclusions

In summary, molecular docking and molecular dynamics revealed the potential of CLOZ as a multifunctional therapeutic agent. MDS studies highlighted the strong binding affinity of CLOZ with COX-2, 5-LOX, AChE, and BuChE and considerable binding affinity for 15-LOX, suggesting its potential to modulate key pathways associated with neuroinflammation and neurodegeneration. In addition to that, CLOZ also demonstrated binding affinity with Caspase-3 in MDS. The biochemical analysis of brain samples from LPS- and CLOZ-treated rats demonstrated that CLOZ may exert neuroprotective effects in an LPS-induced neurotoxicity model. These effects include suppression of inflammatory response, improvement in the cholinergic system, and protection against apoptotic as well as oxidative stress pathways. The observed reductions in COX-2, 5-LOX, AChE, NF-κB, TNF-α, IL-6, Caspase-3, and MDA, as well as the improvement in the levels of IL-10 and TGF-β1, underscore its potential as a neuroprotective agent with anti-inflammatory, anti-apoptotic, and antioxidant properties. These findings provide a compelling basis for further exploration of CLOZ as a candidate for treating neurodegenerative diseases, particularly AD, where its ability to target multiple pathological mechanisms could offer substantial therapeutic benefits. However, the study has certain limitations, which include the absence of in vitro evaluations to validate its activity against the target enzymes. Addressing this gap would strengthen the case for advancing CLOZ with an aim toward clinical investigations.

## Figures and Tables

**Table 1:** Predicted binding affinity (kcal/mol) of the co-crystallized ligand and CLOZ.

Sr. No.	Ligands	AChE	BuChE	COX-2	5-LOX	15-LOX	Caspase-3
1	Co-CL	12.1	10.9	9.1	9.7	5.8	8.2
2	CLOZ	9.1	9.2	7.9	7.6	8.8	6.7

**Table 2:** Root mean square deviation (RMSD) values and standard deviation (SD) values for backbone atoms and ligands calculated across a simulation run of 150 ns.

	Enzyme	AChE	BuChE	COX-2	5-LOX	15-LOX	Caspase-3
Backbone	RMSD (Avg)	0.25	0.25	0.33	0.21	0.22	0.31
	SD	0.02	0.02	0.06	0.03	0.02	0.03
Ligand	RMSD (Avg)	0.30	0.20	0.11	0.27	0.22	0.26
	SD	0.09	0.07	0.05	0.09	0.06	0.10

**Table 3:** Radius of gyration (Rg, nm) average values, standard deviation, and standard error of mean values for all proteins complexed with CLOZ.

	AChE	BuChE	COX-2	5-LOX	15-LOX	Caspase-3
Rg (Avg)	2.34	2.35	2.49	2.82	2.83	1.82
SD	0.01	0.01	0.01	0.02	0.01	0.01
SEM	0.000172	0.000237	0.000366	0.000427	0.000279	0.000319

## Data Availability

The data presented in this study are available from the corresponding author upon reasonable request. The data are not publicly available due to privacy issues.
